# UVA-activated riboflavin promotes collagen crosslinking to prevent root caries

**DOI:** 10.1038/s41598-018-38137-7

**Published:** 2019-02-04

**Authors:** R. Uemura, J. Miura, T. Ishimoto, K. Yagi, Y. Matsuda, M. Shimizu, T. Nakano, M. Hayashi

**Affiliations:** 1 Department of Restorative Dentistry and Endodontology, Graduate School of Dentistry, Osaka University, Osaka, Japan; 2 Division for Interdisciplinary Dentistry, Graduate School of Dentistry, Osaka University, Osaka, Japan; 3 Division of Materials and Manufacturing Science, Graduate School of Engineering, Osaka University, Osaka, Japan; 4 Division of Clinical Cariology and Endodontology, Graduate School of Dentistry, Health Sciences University of Hokkaido, Hokkaido, Japan

## Abstract

Root caries is an increasingly problem in aging societies with severe implications for the general health and wellbeing of large numbers of people. Strengthening type-I collagen, a major organic component of human dentin, has proved effective in preventing root caries. This study sought to determine whether exposure to riboflavin followed by UVA irradiation (RF/UVA) could promote additional collagen crosslinking, and thus improve the acid and enzymatic resistance of human dentin under simulated oral environments. If so, it could offer potential for treatment of the intractable problem of root caries. The greatest flexural strengths were found in dentin exposed to a 0.1% riboflavin solution for 1 minute followed by 1,600 mW/cm^2^ UVA irradiation for 10 minutes. Mineral loss and lesion depth were significantly lower in the RF/UVA group than in the control group. The microstructures of dentinal tubules and collagen networks after RF/UVA treatment retained their original forms after acidic and enzymatic degradation. In conclusion, RF/UVA treatment may be a new method for preventing root caries with promising prospects for clinical application.

## Introduction

Root caries is an important issue, especially in aging societies^1–3^. In elderly people, root surfaces are frequently exposed either because of age-related gingival recession, or as a result of periodontal therapy. In addition, the salivary flow tends to decrease with aging, often exacerbated because of systemic medications^4^. Consequently, dental biofilms can easily adhere and accumulate on exposed root surfaces and initiate root carious lesions. The critical pH of root surfaces that initiates demineralization is significantly higher than that of enamel^5–7^. This means that once a demineralized root surface develops a cavity, the carious lesions swiftly expand and penetrate proximal and sub-gingival lesions along the cement-enamel junction even without causing severe pain^4,8^. Clinicians face difficulties in restoring such expanded root carious lesions because of limited accessibility and poor moisture control. Therefore, the prevention of root caries before cavity formation is critically important for promoting the life-long oral health of elderly people. Good oral health is essential for good general health and wellbeing.

There are two stages in the development of root caries. Initially, inorganic substances such as hydroxyapatite are dissolved by acid from bacteria; then, the demineralized dentin matrix, including type-I collagen, which works as a scaffold for mineral deposition, is degraded by internal enzymes, causing further mineral loss^9–13^. How can we prevent this destructive path? Our idea was that strengthening type-I collagen network, a major organic component of dentin, could be effective in preventing root caries^14–17^.

We have previously reported that the mechanical strength of human dentin could be increased approximately two-fold after ultraviolet (UV) irradiation and the consequent newly-formed collagen crosslinking^18^. In the field of ophthalmology, exposure to riboflavin (RF) followed by UVA irradiation (RF/UVA) has already been clinically applied successfully in keratoconus^19–22^. RF (vitamin B2), used as a photosensitizer, produces active oxygen radicals after excitation by UVA, which promotes collagen crosslinking. In dentistry, RF/UVA treatment of demineralized dentin after acid etching was reported to be effective in increasing the bond strength of composites^23–25^. It was also shown that RF/UVA treatment improved the strength of human dentin^26^. However, the optimal parameters for the use of RF/UVA treatment to maximize the strength of human dentin have not been fully determined. Nor, until this study, has there been a full investigation whether dentin strengthened by RF/UVA treatment can effectively prevent demineralization by inhibiting the degradation of collagen.

The purpose of this study, therefore, was to investigate, under simulated oral environments, whether RF/UVA treatment improves the acid and enzymatic resistance of human dentin by inducing additional crosslinking.

## Results

**Figure 1:**
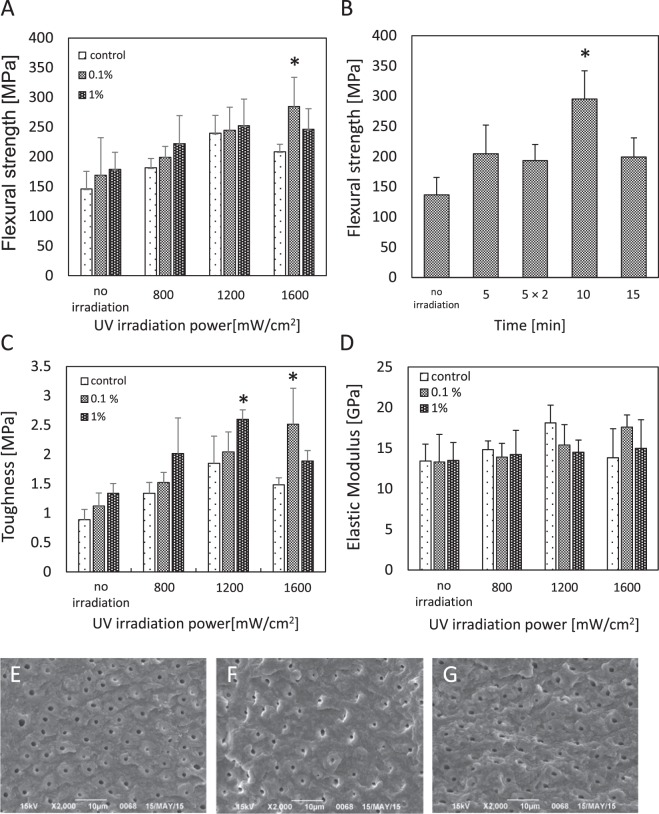
Effects of riboflavin/ultraviolet light (RF/UVA) treatment on the mechanical characteristics of human dentin. (**A**) Flexural strength of human dentin after RF/UVA treatment. (**B**) Effect of UV irradiation time on flexural strength of human dentin (UVA: 1,600 mW/cm^2^, 0.1% RF for 1 min). (**C**) Toughness of human dentin after RF/UVA treatment. (**D**) Elastic modulus of human dentin after RF/UVA treatment. *Statistically significant differences were detected compared to control. (One or two-way ANOVA and Scheffe’s F test, *P* < 0.05, n = 5–7). (**E**–**G**) Scanning electron microscope images of fractured surfaces after a flexural test. (**E**) Control group, (**F**) UVA 1,600 mW/cm^2^ for 10 min, (**G**) 0.1% RF for 1 min + UVA 1,600 mW/cm^2^ for 10 min. The dentin in the control group (**E**) showed a flat fracture surface with clear microstructures, while the specimens subjected to the UVA (**F**) and RF/UVA (**G**) treatments showed rougher surfaces with gaps in the peritubular dentin.

After RF/UVA treatment, the greatest flexural strengths were found in specimens exposed to an 0.1% RF solution for 1 minute followed by 1,600 mW/cm^2^ UVA irradiation for 10 minutes (295.3 ± 46.6 MPa) (Fig. 1A,B). The flexural strength reached approximately 2.2 times the level in the control group (136.6 ± 29.0 MPa). A similar tendency was observed for toughness (Fig. 1C). There were no significant differences in elastic modulus across the testing conditions (Fig. 1D). Based on the fractographic observations, the fracture surfaces in the control group appeared to be smooth (Fig. 1E), while in the UVA irradiation and RF/UVA groups, the surfaces showed gaps in peritubular dentin (Fig. 1F,G), indicating that a larger fracture energy was needed compared to the controls. Based on the overall results, the most effective parameters for strengthening human dentin were exposure to a 0.1% RF solution for 1 minute followed by 1,600 mW/cm^2^ UVA irradiation for 10 minutes. These parameters were used for further studies.

**Figure 2:**
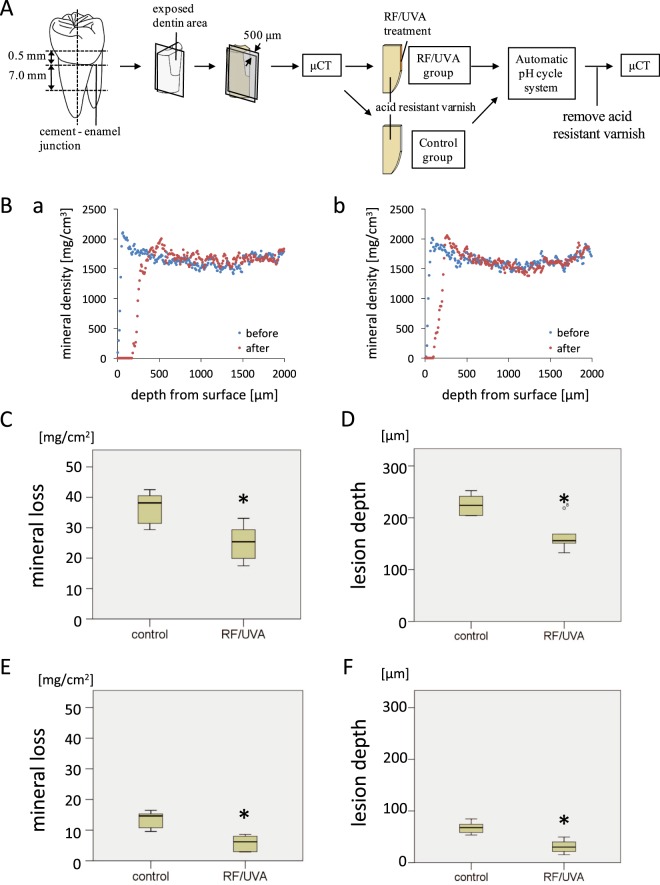
Acid resistance of human dentin after riboflavin/ultraviolet light (RF/UVA) treatment. (**A**) Preparation of root dentin specimens. (**B**) Micro-computed tomography profiles of dentin in control (a) and RF/UVA (b) groups before and after pH-cycling. (**C**–**F**) Mineral loss (**C**,**E**) and lesion depth (**D**,**F**) of demineralized dentin. (**C**,**D**) Show the results after treatment with a demineralizing cycling (pH 4.5 for 2 min, interval of 3 min, pH 6.8 for 60 min, 6 cycles per day), while (**D**,**E**) show the results after remineralizing cycling (pH 4.5 for 2 min, pH 6.8 for 30 min, 3 cycles per day). *Statistically significant differences were detected between the groups. (Student’s t-test, *P* < 0.05, n = 7)

The pH-cycling tests showed that RF/UVA treatment significantly reduced the amount of mineral loss compared to the control group, for both demineralizing and remineralizing cycles. The mineral loss in the RF/UVA group was 25.1 ± 6.2 mg/cm^2^, while that in the control group was 36.7 ± 5.3 mg/cm^2^ (Fig. 2C). The lesion depth in the RF/UVA group (163.8 ± 26.9 µm) was also significantly smaller than that in the control group (225.1 ± 18.0 µm) (Fig. 2D). Milder demineralization was found for the remineralizing cycle than for the demineralizing cycle, in terms of both mineral loss and lesion depth. The mineral loss in the RF/UVA group was 5.7 ± 2.6 mg/cm^2^, while that in the control group was 13.3 ± 2.8 mg/cm^2^ (Fig. 2E). The lesion depth in the RF/UVA group (31.3 ± 12.0 µm) was also significantly smaller than that in the control group (67.4 ± 10.8 µm) (Fig. 2F).

**Figure 3:**
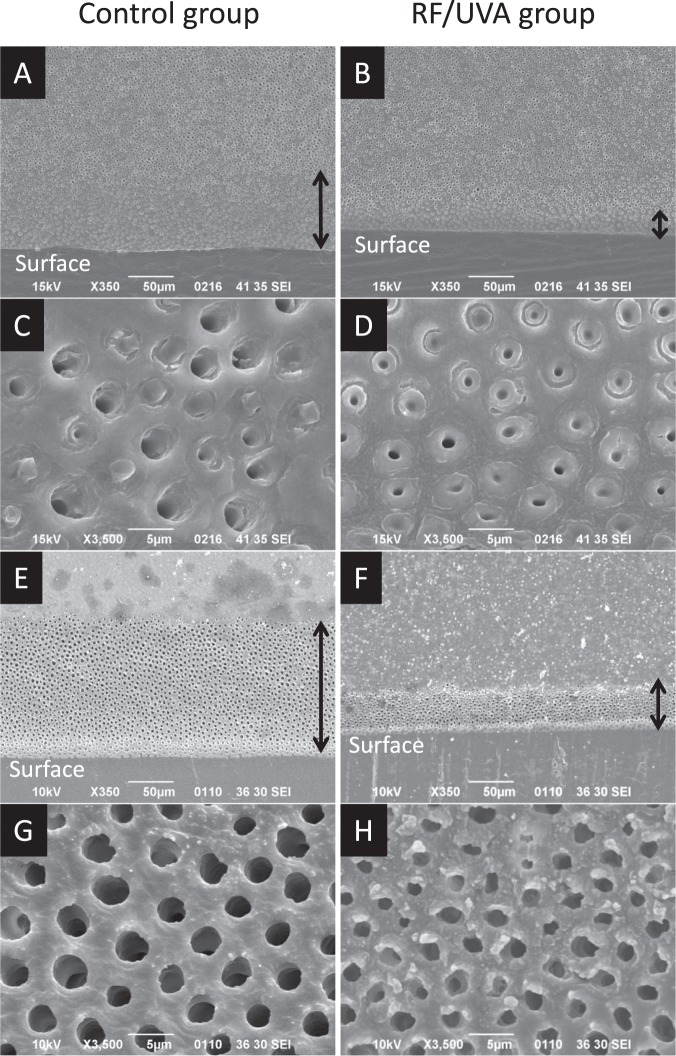
Scanning electron microscope images of demineralized dentin surfaces. Samples were immersed in a demineralizing solution of pH 5.0 (**A**–**D**) and 10% ethylenediaminetetraacetic acid (EDTA) (**E**–**G**) for 3 days each. (**A**,**C**,**E**,**G**) control group; (**B**,**D**,**F**,**H)** riboflavin/ultraviolet light (RF/UVA) treated group. The depth of the demineralized zone (black arrow) was clearly larger in the control (**A**,**E**) compared to that in the RF/UVA group (**B**,**F**). Dentinal tubules in the demineralized zone were enlarged in the control group (**C**,**G**), while the demineralization was less aggressive in the RF/UVA group (**D**,**H**).

Figure 3 shows SEM images of the demineralized dentin after and without RF/UVA treatment. The images demonstrate that the depth of demineralization in the RF/UVA group was smaller than that in the control group (Fig. 3A,B with the demineralizing solution, 3E and 3 F using 10% EDTA). When the demineralizing solution (pH 5.0) was used, the demineralization depth in the RF/UVA group was 27.8 ± 2.8 μm, which was significantly smaller than that in the control group (74.8 ± 7.5 μm) (*P* < 0.05). Dentinal tubules in the demineralized zone were clearly enlarged in the control group (Fig. 3C,G), whereas the demineralization was less aggressive in the RF/UVA group (Fig. 3D,H).

**Figure 4:**
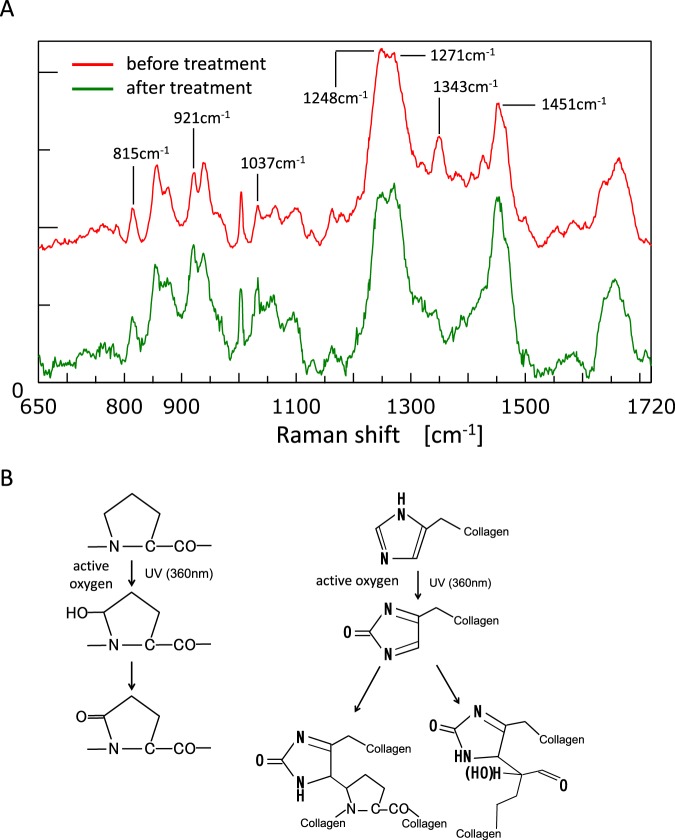
Chemical changes in the structure of dentin collagen after riboflavin/ultraviolet light (RF/UVA) treatment. (**A**) Laser Raman spectra of dentin collagen before and after RF/UVA treatment. 921 and 1,037 cm^−1^: a C-C bond in a proline ring, 1,248 and 1,271 cm^−1^: CN-stretch and NH-deformations, 1,343 and 1,451 cm^−1^: wagging and deformation of CH_2_ and CH_3_. (**B**) Possible structural changes in dentin collagen after RF/UVA treatment. First, active oxygen is released from an excited collagen molecule as a result of RF/UVA treatment. Second, singlet oxygen in a high energy state creates covalent bonds between hydroxyproline or proline residues and histidine residues in adjacent chains. These form new inter- and intra-molecular collagen crosslinks.

We next compared the Raman spectra before and after RF/UVA treatment using 815 cm^−1^ peak as a reference indicating the vibration in the mainframe structure of the collagen^27^. The results showed that the Raman spectra of the collagen were amplified after RF/UVA treatment at peaks of 921 and 1,037 cm^−1^, indicating a C-C bond in a proline ring. In addition, peaks at 1,248 and 1,271 cm^−1^, indicating CN-stretching and NH-deformation, and at 1,343 and 1,451 cm^−1^, indicating wagging and deformation of CH_2_ and CH_3_, also indicated altered structures (Fig. 4A).

**Figure 5:**
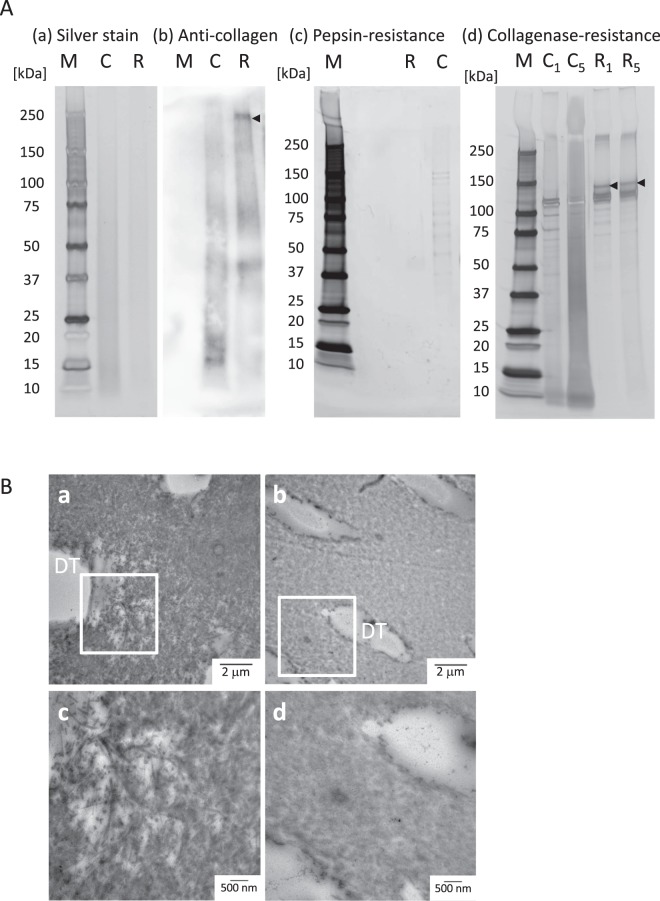
Enzymatic resistance of dentin collagen after riboflavin/ultraviolet light (RF/UVA) treatment. (**A**) SDS-PAGE and western blotting of dentin collagen after RF/UVA treatment. M: marker, C: control group, R: RF/UVA treatment group, C_1_, C_5_: control group, treated by collagenase for 1 or 5 days, R_1_, R_5_: the RF/UVA group, treated by collagenase for 1 or 5 days. The silver staining shows there was no difference in total protein between the control and the RF/UVA groups (a). The anti-collagen antibody staining shows that type-I collagen with high molecular weights were present in the RF/UVA group (black arrow), whereas collagen with lower molecular weights was predominant in control group (b). After treatment with pepsin, the resulting profile shows that dissolved collagen was observed in the control group, while no clear profile appeared in the RF/UVA group (c). After treatment by collagenase for 1 and 5 days, the profiles show that dissolved collagen were detected in the control group (d). After 5-day treatment by collagenase, highly dissolved collagen appeared as a contiguous belt in the control group, while the collagen profiles after RF/UVA treatment showed clear bands in the area of 100–120 kDa. Full-length gels and blots are presented in Supplementary Figure S1. (**B**) TEM images of dentin specimens degraded by collagenase for 5 days. a and c: control group; b and d: RF/UVA group. Areas indicated with white squares in a and c are shown magnified in c and d. DT: dentinal tubules. The collagen around dentinal tubules was severely degraded in the control group after degradation (a,c), whereas in the RF/UVA group, the collagen retained its original form (b,d).

The results of the SDS-PAGE analysis are summarized in Fig. 5A. There were no differences between the control and the RF/UVA groups in the total amount of protein, as demonstrated in the silver-staining (Fig. 5A-a). However, western blotting using an anti-collagen antibody showed that type-I collagen with high molecular weight appeared for the RF/UVA group; while collagen with lower molecular weight was predominant in the control group (black arrow in Fig. 5A-b). This suggested that RF/UVA treatment induced collagen crosslinking, resulting in the formation of larger molecules. The profile after pepsin treatment suggested that dissolved collagen was present in the control group, while no clear profile appeared in the RF/UVA group (Fig. 5A-c). After treatment with collagenase for one and five days, the profiles indicated dissolved collagen in the control group (Fig. 5A-d). After five-day treatment with collagenase, highly dissolved collagen appeared as a contiguous belt for the control group. After RF/UVA treatment, on the other hand, the collagen profiles showed clear bands in the areas of 100–120 kDa, indicating collagen α1 and 2 chains. This demonstrated the higher enzymatic resistance of dentin collagen after RF/UVA treatment.

TEM images showed that after degradation with collagenase for five days, collagenous fiber around the dentinal tubules was severely degraded in the control group (Fig. 5B-a,c), whereas the structure of collagen retained its original in the RF/UVA group (Fig. 5B-b,d).

## Discussion

RF/UVA treatment of human dentin produced two clinically beneficial effects: improvement in its mechanical properties, and prevention of degradation by acids and enzymes as a result of increased collagen crosslinking. Research has shown that the stable structures of collagen matrix are important for mineralization of dentin and stabilization of its mechanical strength^14,16,17^. When the dentin collagen is degraded as a carious lesion progresses, the acid permeates the porous dentin, causing further mineral loss. On the other hand, newly developed crosslinks after RF/UVA treatment promoted resistance against the degradation by acid and enzyme. In addition, the dentin with crosslinks after RF/UVA treatment demonstrated superior mechanical properties. These findings suggested that RF/UVA treatment might be clinically effective for preventing root caries as well as root fracture.

In recent years, various collagen crosslinking agents such as proanthocyanidins, glutaraldehydes, and carbodiimides have been investigated^28–31^. RF, a water-soluble vitamin, is contained in daily foods, and has excellent biocompatibility without any hazards to oral health; while glutaraldehydes and carbodiimides, which are artificially generated substances, demonstrated serious toxicity if taken orally^32–34^. Proanthocyanidins, which are plant-derived polyphenol extracts, were reported to increase the mechanical strength of dentin, improve its adhesive properties, and inhibit the degradation of collagen^17,31,35,36^. However, they had the disadvantage of causing discoloration of dentin^34,37–39^. RF solutions also exhibited yellowish colors, but at clinically acceptable levels of discoloration; and the yellowish coloration mostly disappeared after the UVA treatment.

The optimal parameters for RF/UVA treatment of human dentin were identified as exposure to a 0.1% RF solution followed by 1,600 mW/cm^2^ UVA irradiation for 10 minutes. A previous study showed the maximum concentration of the RF solution was limited to 1.0%, because solutions with over 1.0% RF saturated easily, and powdery RF was precipitated on the dentin surface after the UVA irradiation^40^. This precipitated RF inhibited the crosslinking effect of the UVA irradiation. In addition, RF solutions at high concentrations cause strong yellowish discoloration of dentin^23,24^.

Lower flexural strength was observed in the group treated with 1% RF solutions followed by irradiation with 1,600 mW/cm^2^ UVA (Fig. 1A), probably due to concentration quenching. Moreover, the flexural strength also decreased after exposure to UVA for longer durations (Fig. 1A control group and Fig. 1C). These findings suggest that over-exposure to UVA may cause the deterioration of dentin collagen. Therefore, it is essential to control both the intensity and the duration of the UVA irradiation to maximize the crosslinking of dentin collagen.

The value in this case of *in vitro* research was to set the optimal parameters for *in situ* studies, which would be problematical without such ground work. In the present study, we used an automatic pH-cycling system that enabled good simulation of pH fluctuations in an oral cavity^41^. We set two conditions for the demineralizing and remineralizing cycles, to represent the oral cavities of individuals with high and low caries risk (Fig. 2). Frequent cavitation was found on the root surface after the demineralizing cycle, and subsurface lesions were found after the remineralizing cycle. These typical morphologies of root carious lesions indicated that the pH-cycling used in the present study offered good simulations of different caries risks in clinical conditions.

SEM images of root dentin treated by acid and EDTA demonstrated that strengthening the collagen network by RF/UVA treatment was effective in preventing the outflow of hydroxyapatite crystals around dentinal tubules. The images at high magnification clearly showed that the exposed dentinal tubules in the control group were significantly enlarged compared with those in the RF/UVA treatment group (Fig. 3). This suggests that strengthening the collagen network can help in preventing incipient mineral loss, which is an initial step in developing a carious lesion. In addition, the results of western blotting and TEM observation (Fig. 5A,B) demonstrated that the degradation of collagen around dentinal tubules was inhibited by RF/UVA treatment. Therefore, RF/UVA treatment was proved to be a novel method to prevent root caries by promoting additional crosslinking, which is effective to keep hydroxyapatite crystals even after the exposure of enzyme.

Raman spectroscopic analysis showed the characteristic accentuation in both amide-III bands and proline imide ring that are associated with crosslinking (Fig. 4A), which is consistent with previous reports of type-I collagen crosslinking induced by riboflavin^24^. The likely mechanism of additional collagen crosslinking is illustrated in Fig. 4B ^22,42^. First, active oxygen is released from excited collagen molecules by RF/UVA treatment. Second, singlet-oxygen with high energy status induces covalent bonds between hydroxyproline or proline residues and histidine residues in adjacent chains.

Based on the results of the present study, RF/UVA treatment could have promising potential applications for root dentin to prevent root caries as the dentin pre-treatment. In conclusion, RF/UVA treatment promotes collagen crosslinking and may offer a potential new method for preventing root caries.

## Materials and Methods

### Preparation of dentin specimens

Human third molars free of caries were stored in Hanks’ balanced salt solution (HBSS) at 4 °C and used within three months of extraction. All experiments are carried out in accordance with protocols approved by the Research Ethics Committee of the Faculty of Dentistry, Osaka University (H25-E28). Informed consent was obtained from all patients, aged 22 to 39 years old, who supplied the teeth.

### Identification of optimal parameters for RF/UVA treatment

Dentin specimens were prepared using a low-speed diamond saw (Isomet2000, Buehler, USA). Disk and beam-shaped specimens were prepared. Disk-shaped specimens with approximately 1.0 mm thickness were obtained from coronal central portions of the molars, by sectioning them perpendicular to the tooth axis. Beam-shaped specimens, measuring approximately 1.7 × 0.2 × 8.0 mm, were obtained from the cement-enamel junctions of the molars. The dentinal tubules in the beam-shaped specimens were set up to run parallel to the loading surface along the specimen length.

RF solutions were prepared by dissolving riboflavin-5-monophosphate sodium (Tokyo Chemical Industry, Japan) in distilled water. The solutions were stored in lightproof test tubes to avoid any light-activation. The dentin specimens were immersed in RF solutions (0.1 or 1%) for 1 min, gently air-dried, then exposed to UVA light at a 365 nm wavelength in a UVA light emitting diode (LED) irradiation unit (ZUV-C30H, Omron, Japan). The distance from the UVA light source to the specimen was approximately 10 mm.

To identify the optimal parameters for RF/UVA treatment, flexural fracture testing was performed after UVA irradiation with varying intensities (800, 1,200, 1,600 mW/cm^2^) and durations (5, 10, 15 minutes). Flexural fracture testing was conducted on the beam-shaped specimens (n = 6 per group) using a three-point bending geometry in a universal testing instrument (Autograph AG-IS, Shimadzu, Japan), with a crosshead speed of 0.1 mm/min. The flexural strength (σ, N/mm^2^) and toughness (*u*, N/mm^2^) was calculated with the method described by Shinno *et al*.^43^. Elastic modulus (GPa) was also calculated from the stress–strain curve of the flexural testing.

Fractured surfaces of all specimens were then sequentially dehydrated in ethanol concentrations of 50, 70, 80, 90, 95 and 100% and dried in a desiccator for 24 hours. After sputter-coating with platinum, the specimens were observed by scanning electron microscopy (SEM) (JSM-310, JEOL, Japan) at magnification 2000x.

### Assessment of dentin acid resistance after RF/UVA treatment

Dentin specimens were prepared as previously reported (Fig. 2A)^44^. Root dentin specimens were sectioned perpendicularly to the axis, at points 0.5 mm above and 7.0 mm below the cement-enamel junction (CEJ). The buccal half of the block was used for the experiment. The surface of the root block was cut until the dentin was exposed. The root block was then sliced in two longitudinally, measuring approximately 500 μm in thickness at the center of the exposed dentin surface.

The surfaces of the root dentin specimens, other than the exposed dentin area (0.5 × 2 mm), were covered with an acid-resistant varnish, to allow demineralization only from the exposed dentin.

**Figure 6:**
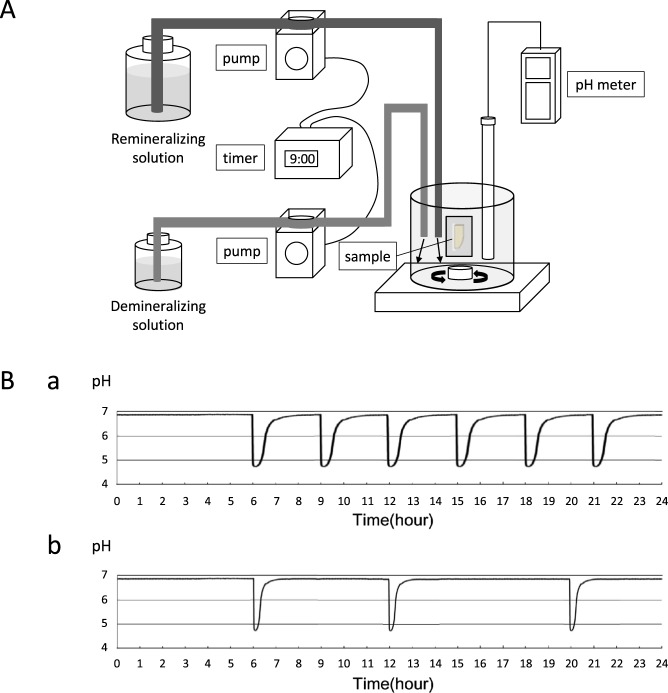
Schematic diagram of automatic pH-cycling system. (**A**) Schema of the pH-cycling system. Demineralizing and remineralizing solutions were pumped into a plastic beaker with two delivery pumps controlled by a programmable timer. (**B**) Twenty-four-hour pH fluctuation patterns. (a) Demineralizing cycle (simulating a high carious risk condition); six cycles per day (at 06:00, 09:00, 12:00, 15:00, 18:00, and 21:00). The average time for the pH to reach 5.5 or less during each cycle was 30 min, and the average time to return to the initial pH was 50 min. (b) Remineralizing cycle (simulating a low carious risk condition); three cycles per day (at 06:00, 12:00, and 18:00). The average time for the pH to reach 5.5 or less during each cycle was 8 min, and the average time to return to the initial pH was 23 min.

To simulate the daily pH changes in an oral cavity, demineralization tests were conducted using an automatic pH-cycling system, with the method described by Matsuda *et al*. (Fig. 6A)^41^. Two pH cycle patterns were applied, representing high and low carious risk patients (Fig. 6B). For a high carious risk condition, a demineralizing cycle was set, in which the cycle began with a demineralizing solution (0.2 M lactic acid, 3.0 mM CaCl_2_, 1.8 mM KH_2_PO_4_, pH 4.5) for 2 min, followed by an interval of 3 min, then replaced with a remineralizing solution (0.02 M HEPES, 3.0 mM CaCl_2_, 1.8 mM KH_2_PO_4_, pH 6.8) for 60 min. The average time at which the pH reached 5.5 or less during each cycle (demineralization time) was 30 min, and the average time to return to the initial pH (recovery time) was 50 min. There were six cycles per day (06:00, 09:00, 12:00, 15:00, 18:00, and 21:00). To simulate a low carious risk condition, a remineralizing cycle was used, in which the cycle began with a demineralizing solution for 2 min, followed by a remineralizing solution for 30 min. The average demineralization time was 8 min, and the average recovery time was 23 min. There were three cycles per day (06:00, 12:00, and 18:00). After demineralization, the exposed dentin area was washed with distilled water and the acid-resistant varnish was removed.

Before and after demineralization by pH-cycling, all specimens were scanned with a micro-computed tomography (μCT) system (inspeXio SMX-100CT, Shimadzu, Japan). Data were acquired at 512 × 512 pixel resolution, with 9.0 μm isotropic voxels. A series of reference phantoms included 4 hydroxyapatite disks with different concentrations (100, 200, 300, 400 mg/cm^3^), and an aluminum pole (1,550 mg/cm^3^) was also scanned for calibration.

Two-dimensional images of the specimens were reconstructed using CT-analysis software (CT-solver, Shimadzu, Japan). The resulting μCT images were analyzed using image analysis software (Image J, NIH, USA) to produce an overall mineral profile of the demineralized zone 1 mm from the top edge of the sample (Fig. 2B). The lesion depth (μm) was defined as the distance from the baseline of the sound dentin surface (an intact edge covered by acid-resistant varnish) to a lesion front at which the mineral content was 95% of the value for sound dentin. Mineral loss (mg/cm^2^) was calculated by integrating the differences in mineral content before and after demineralization over the volume of the specimen.

### Investigation of demineralized dentin microstructures after RF/UVA treatment

Two sections were taken from one beam-shaped specimen, by cutting along the axis to obtain mirror images. One section was subjected to RF/UVA treatment, while the other was used as a control. After immersion in a demineralizing solution (pH 5.0) or 10% ethylenediaminetetraacetic acid (EDTA) for three days (n = 4 per group), the morphologies of both surfaces were observed with SEM. The depth of the demineralized zone from the surface layer was measured using image analysis software (Image J).

### Investigation of chemical changes in the structure of dentin collagen after RF/UVA treatment

Disk-shaped specimens (n = 5) were demineralized in 10% EDTA (pH 7.4) for 7 days to expose dentin collagen. Raman spectra of the demineralized dentin were analyzed before and after RF/UVA treatment, by laser Raman microscopy (RAMAN touch, Nanophoton, Japan). The laser wavelength was 785 nm, and the accumulation time was 100 seconds. Excited Raman scattering light was collected with the same objective lens and guided to the spectrograph, which had a focal length of 500 mm. The Raman light was then recorded with a thermoelectrically cooled CCD camera as the Raman spectrum, which contained chemical bond information.

### Evaluation of acid and enzymatic resistance of dentin collagen after RF/UVA treatment

Columns of diameter 1 mm were punched out from root dentin, with or without RF/UVA treatment. Some of the resultant columns were solubilized in 1 M hydrochloric acid for 1 day. Other columns were demineralized in 10% EDTA (pH 7.4) for 7 days and then dissolved in pepsin from porcine gastric mucosa (0.05 mg/mL in 0.5 M acetic acid, Sigma-Aldrich, USA) for 1 day at 37 °C, or collagenase from *Clostridium histolyticum* (0.2 mg/mL, Sigma-Aldrich) for 1 or 5 days at 37 °C.

Cold acetone was poured to precipitate the solution, and the suspension was centrifuged at 10,000 rpm for 20 min. The resulting precipitant was dissolved in phosphate-buffered saline (PBS) and maintained at 4 °C. An aliquot solution was diluted in Laemmli sample buffer and electrophoresed using sodium dodecyl sulfate–polyacrylamide gel electrophoresis (SDS-PAGE)^45^. Some gels were silver-stained for protein detection, and the others were used for western blotting.

The protein was transferred to a polyvinylidene difluoride (PVDF; Bio-Rad, USA) membrane in a semi-dry blotter. The PVDF membranes were treated with a blocking agent (Ez Block Chemi, ATTO, Japan), then immunostained with primary antibodies (rabbit anti-collagen polyclonal antibodies). The immunocomplexes were detected with a chemiluminescent method (EzWest Lumi Plus, ATTO, Japan; GeneGnome-5 system, Syngene, UK) after incubation with a secondary antibody^46^.

Root dentin specimens were fixed in 2.5% glutaraldehyde after degradation by collagenase for 5 days, and dehydrated in a graded series of aqueous ethanol solutions, then embedded in epoxy resin. Sections of 70 nm thickness were prepared using an ultramicrotome and examined with a transmission electron microscope (TEM) (H800, Hitachi, Japan) at magnifications of 9,000× and 20,000×.

### Statistical analysis

Statistical analysis of the flexural strength results was accomplished by one and two-way analysis of variance (ANOVA), with Scheffe’s *F* test. In the μCT analysis, statistical differences were evaluated with Student’s t-test. Differences with *P* < 0.05 were considered significant.

## Supplementary information

Supplementary Figure S1
