# Supplementary material for: UVA-activated riboflavin promotes collagen crosslinking to prevent root caries
Source: Sci Rep. 2019 Feb 4;9:1252. doi: 10.1038/s41598-018-38137-7 (PMC6362121; doi:10.1038/s41598-018-38137-7)
Supplement: Supplementary file 1 — Supplementary Figure S1 [file 41598_2018_38137_MOESM1_ESM.pdf]

Supplementary Information

UVA-activated riboflavin promotes collagen crosslinking to prevent root caries

R. Uemura[1*], J. Miura[2], T. Ishimoto[3], K. Yagi[1], Y. Matsuda[4], M. Shimizu[2], T. Nakano[3], M. Hayashi[1]

[1] Department of Restorative Dentistry and Endodontology, Graduate School of Dentistry, Osaka University, Osaka, Japan

[2] Division for Interdisciplinary Dentistry, Graduate School of Dentistry, Osaka University, Osaka, Japan

[3] Division of Materials and Manufacturing Science, Graduate School of Engineering, Osaka University, Osaka, Japan

[4] Division of Clinical Cariology and Endodontology, Graduate School of Dentistry, Health Sciences University of Hokkaido, Hokkaido, Japan

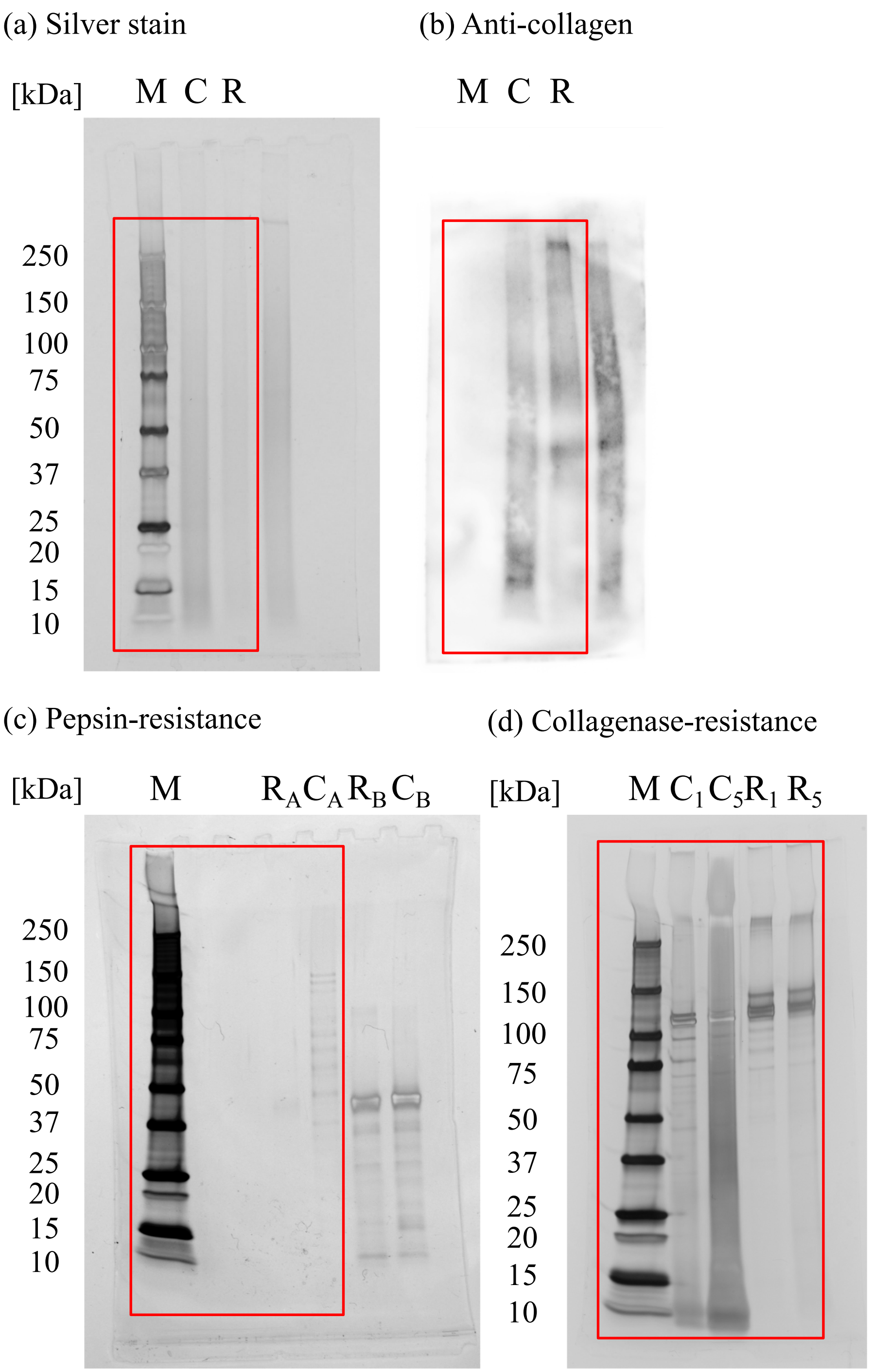

Supplementary Figure S1. Full-length gels and blots for Figure 5.
Red squares indicate where the images were cropped.
M: marker, C: control group, R: RF/UVA treatment group, R_A, C_A: appropriate concentration of pepsin solution, R_B, C_B: 10 times higher concentration of pepsin solution, C_1, C_5: control group, treated by collagenase for 1 or 5 days, R_1, R_5: the RF/UVA group, treated by collagenase for 1 or 5 days.
